# Pallidal Recordings in Chronically Implanted Dystonic Patients: Mitigation of Tremor-Related Artifacts

**DOI:** 10.3390/bioengineering10040476

**Published:** 2023-04-15

**Authors:** Jasmin Del Vecchio Del Vecchio, Ibrahem Hanafi, Nicoló Gabriele Pozzi, Philipp Capetian, Ioannis U. Isaias, Stefan Haufe, Chiara Palmisano

**Affiliations:** 1 Department of Neurology, University Hospital of Würzburg and Julius-Maximilian-University Würzburg, 97080 Würzburg, Germany; hanafi_i@ukw.de (I.H.); pozzi_n2@ukw.de (N.G.P.); capetian_p@ukw.de (P.C.); isaias_i@ukw.de (I.U.I.); palmisano_c@ukw.de (C.P.); 2 Centro Parkinson e Parkinsonismi, ASST G. Pini-CTO, 20122 Milano, Italy; 3 Uncertainty, Inverse Modeling and Machine Learning Group, Technische Universität Berlin, 10623 Berlin, Germany; stefan.haufe@charite.de; 4 Physikalisch-Technische Bundesanstalt Braunschweig und Berlin, 10587 Berlin, Germany; 5 Berlin Center for Advanced Neuroimaging, Charité—Universitätsmedizin Berlin, 10117 Berlin, Germany

**Keywords:** dystonia, tremor, local field potentials, globus pallidus, deep brain stimulation

## Abstract

Low-frequency oscillatory patterns of pallidal local field potentials (LFPs) have been proposed as a physiomarker for dystonia and hold the promise for personalized adaptive deep brain stimulation. Head tremor, a low-frequency involuntary rhythmic movement typical of cervical dystonia, may cause movement artifacts in LFP signals, compromising the reliability of low-frequency oscillations as biomarkers for adaptive neurostimulation. We investigated chronic pallidal LFPs with the Percept^TM^ PC (Medtronic PLC) device in eight subjects with dystonia (five with head tremors). We applied a multiple regression approach to pallidal LFPs in patients with head tremors using kinematic information measured with an inertial measurement unit (IMU) and an electromyographic signal (EMG). With IMU regression, we found tremor contamination in all subjects, whereas EMG regression identified it in only three out of five. IMU regression was also superior to EMG regression in removing tremor-related artifacts and resulted in a significant power reduction, especially in the theta-alpha band. Pallido-muscular coherence was affected by a head tremor and disappeared after IMU regression. Our results show that the Percept PC can record low-frequency oscillations but also reveal spectral contamination due to movement artifacts. IMU regression can identify such artifact contamination and be a suitable tool for its removal.

## 1. Introduction

Dystonia is a movement disorder characterized by patterned, directional, and often sustained involuntary muscle contractions that produce abnormal postures or repetitive movements [1]. Tremor can be a basic feature of a dystonic contraction and is reported in up to 87% of patients [2]. One of the most common dystonic tremors is head tremor in patients with cervical dystonia (CD) [3], in which it causes a severe reduction in quality of life.

The pathophysiology of CD and dystonic tremor (DT) is not entirely known. It is still unclear whether tremor in dystonia has its own pathophysiology unrelated to dystonia or shares some mechanisms. It is also still debated whether DT, which is the presence of tremor in a body part affected by dystonia, and tremor associated with dystonia (TAWD), defined as tremor in a body part not affected by dystonia [4], are distinct entities or similar syndromes [5,6]. Previous studies revealed increased intermuscular coherence in DT because of a loss of reciprocal inhibition [7,8,9], indicating that DT shares some dystonia pathophysiology [10,11,12]. Functional imaging studies further showed the involvement of the basal ganglia, thalamus, midbrain, and sensory-motor cortex in DT, similar to dystonia without tremor [13,14]. Recently, there has been increasing evidence of cerebellar involvement and interactions between the cerebellum and basal ganglia in DT and dystonia, and most of the literature converges in supporting the involvement of both basal ganglia-thalamo-cortical and cerebello-thalamo-cortical pathways in dystonia [15,16,17,18,19,20,21,22,23,24,25,26,27]. In particular, the cerebellum has a critical role in the generation and expression of tremor and CD [28]. Neuroimaging studies showed greater activation of the anterior cerebellar regions ipsilateral to the direction of head rotation and decreased activation in the posterior cerebellar regions [29,30]. Patients with CD and head tremor showed higher clinical scores of cerebellar dysfunctions, e.g., ataxia, than those without tremor [28], and dystonic tremor improved after deep brain stimulation (DBS) of the cerebellar thalamus (ventralis intermediate nucleus [VIM]) [31]. On the other hand, a recent study comparing patients with CD without tremor, CD with jerky head oscillations, and sinusoidal head oscillations showed a distinct pallidal dysfunction in the group with sinusoidal oscillations [32]. This would favor the hypothesis of a specific contribution of the basal ganglia to the pathophysiology of DT [15].

Chronic DBS of the globus pallidus pars interna (GPi) is a safe and effective treatment for advanced, disabling dystonia [33,34,35,36]. Despite successful results, pallidal stimulation for dystonia remains a poorly standardized therapy with variable clinical outcomes [37]. A significant challenge remains the variability of treatment benefits at an individual level. An added complexity is that improvement after DBS is typically delayed or progressive over months or years [35,38,39].

New implantable devices capable of chronically recording local field potentials (LFPs) during stimulation will enable a better understanding of disease-related brain activity patterns, their evolution over time, and their modulation in response to therapies [40]. This could dramatically improve tailoring treatment to each patient by adapting stimulation parameters (adaptive DBS, aDBS) in response to an input signal that can represent symptoms, motor activity, or other behavioral features [41]. In this regard, two aspects are particularly relevant: (i) the ability to identify robust biomarkers reflecting symptoms and their fluctuations in the context of activities of daily living and (ii) the reliability of the device for real-time monitoring of artifact-free recordings and online adjustment of one or more stimulation parameters [40].

One of the most promising biomarkers for dystonia is an increase in the magnitude of oscillatory activity in the theta-alpha range (3–12 Hz) embedded in GPi-LFPs [42,43,44]. Neumann et al. showed a correlation between these oscillations and the severity of dystonia [42], but their acute suppression might not be followed by a direct change in symptom severity [45]. Additionally, the reliability of this biomarker in chronically stimulated patients has yet to be fully explored. Piña-Fuentes et al. observed a significantly lower theta-alpha frequency power in (still symptomatic) dystonic patients chronically treated with DBS in comparison to newly implanted patients, even when stimulation was suspended [45].

The aim of the present study was to investigate pallidal low-frequency oscillatory activity in our first series of dystonic patients chronically stimulated with the Percept^TM^ PC device (Medtronic PLC). This is one of the first commercially available devices for chronic DBS able to continuously record LFP in real time and transmit them wirelessly to a storage device (a tablet-user interface) [46].

In this study, we particularly focused on dystonic tremor as a possible source of artifactual contamination of GPi-LFP recordings. Indeed, consistent and rhythmic movements are particularly likely to contaminate the power spectrum [47]. This would be critical in subjects with CD as they may present head tremor at a frequency of 1–6 Hz, which falls in the same range as the GPi-LFP’s clinically relevant spectral power [48,49].

## 2. Materials and Methods

### 2.1. Subjects, Surgery, and Clinical Evaluation

We reviewed data collected from eight patients with idiopathic dystonia implanted in the GPi with the Percept PC device (Medtronic, PLC), who were routinely evaluated at our center. Demographic and clinical data are listed in Table 1. Four patients had CD, three had myoclonus dystonia (DM), and one had segmental dystonia primarily affecting the head and the (left and right) arm (MFD). Five of these patients showed dystonic tremors of the head. This was defined as a spontaneous oscillatory, rhythmical head movement [1].

All patients were implanted with standard non-directional DBS leads (3389, Medtronic, PLC). The surgical procedure for DBS implantation has been described previously. Briefly, patients underwent simultaneous bilateral stereotactic implantation of DBS electrodes into the posteroventrolateral internal globus pallidus. The DBS electrode used was model 3389 (Medtronic, PLC), with four platinum-iridium cylindrical contacts of 1.5 mm each and a contact-to-contact separation of 0.5 mm. The DBS electrodes were connected to an implantable pulse generator (IPG) during the same or a subsequent surgery [39,50]. Three out of eight patients received the Percept PC as a battery replacement. Along with GPi-DBS, one patient with DM received DBS of the motor thalamus (i.e., VIM).

We assessed the severity of symptoms with the Toronto Western Spasmodic Torticollis Rating Scale (TWSTRS, severity subscale [51,52]). The evaluations were performed before and after DBS implantation (i.e., on the day of GPi-LFP recordings). After DBS, evaluations were performed in both stimulation-off (stim-off) and stimulation-on (stim-on) conditions with the clinically optimized stimulation parameters.

The local Institutional Review Board approved the study and waived review for the data collection. Informed consent was obtained from all subjects involved in the study according to the Declaration of Helsinki.

### 2.2. Experimental Setup and Recordings

We recorded GPi-LFPs from the chronically implanted electrodes in stim-off condition at least six months after DBS implant. The stimulation was paused for at least 30 min before the experiment. During the recordings, patients were at rest, comfortably sitting in a chair. They were asked to keep their eyes open without speaking or performing any voluntary movement. The average (±standard deviation) recording length was 233.62 s (±141.68 s), ranging from 66.8 s (patient DW02) to 451.48 s (patient DW03). According to the clinical evaluation of a neurologist expert in movement disorders (N.P.), the subject was considered a tremor (T+) or non-tremor (T−) patient. The presence of tremor was further confirmed by inspecting the video recordings of the experiment (VIXTA, BTS).

For one T+ patient (DW02), we performed three additional recordings aimed at investigating the effect of head tremor on LFPs. First, during the execution of an alleviating maneuver (sensory trick or geste antagoniste) [53], namely, a light touch of the face performed with the left hand. This is a peculiar feature of dystonia that enables a temporary relief of dystonic muscle contractions upon sensory stimulation [53,54]. Second, during voluntary alternating (left to right and back) rhythmic movements of the head with small and, third, large amplitude. We limited this second set of recordings to only patient DW02 as she was the only one among the T+ patients with a clinically effective sensory trick.

GPi LFPs were recorded bilaterally from all non-adjacent contact pairs (i.e., 0–3, 0–2, 1–3, where 0 is the lowermost and 3 the uppermost contact, respectively) at a sampling frequency of 250 Hz (*indefinite streaming* mode) [46].

Head tremor was recorded bilaterally with surface electromyography (FREEEMG, BTS) of the sternocleidomastoid and trapezius muscles at a sampling frequency of 1000 Hz. We chose these two muscles because they were affected by tremor in all patients and could be easily recorded. Additionally, we placed one EMG probe on the left chest to record heart activity and remove cardiac artifacts from the LFPs [55] and one on the neck close to the cable connecting the implantable pulse generator (IPG) with the DBS electrodes for synchronization purposes [46,55]. The method for synchronizing LFP and EMG recordings has previously been described in [46,56,57]. Briefly, a transcutaneous electrical nerve stimulation (TENS) burst was delivered at the level of the neck (f = 80 Hz) at the beginning and at the end of each recording session. We used the abrupt drop-off of the TENS artifact simultaneously recorded by the DBS electrodes and EMG probe to align the two signals.

In the T+ group, head tremor was recorded with a triaxial inertial measurement unit (IMU) (Opal, APDM) placed on the forehead at a sampling frequency of 128 Hz. IMU and EMG signals were synchronized by aligning the data with respect to the rising edge of a transistor-transistor logic (TTL) signal going from 0 to 5 V.

### 2.3. Data Analysis

#### 2.3.1. Data Preprocessing

All data were imported to MATLAB (R2022b, The Mathworks, Natick, MA, USA) and analyzed offline using custom codes.

EMG recordings were down-sampled to the LFP sampling frequency, i.e., 250 Hz. Although the IPG was located in the right subclavicular region in five out of eight patients [58], all LFP signals were contaminated by cardiac activity. The cardiac artifacts were removed from the LFP signals by means of singular value decomposition (SVD). We first detected the cardiac QRS peaks as recorded by the EMG probe placed on the chest. We then divided the LFP signals into epochs centered on each QRS peak. For each epoch, we computed the SVD of the LFP signals and visually identified all the eigenvectors corresponding to the ECG artifact. We then reconstructed the cardiac artifact from these components and subtracted it from the raw LFP [55].

To characterize tremor, EMGs were band-pass filtered in the band 1–120 Hz (second order IIR filter). We used the absolute value of the Hilbert transform of the EMG signals for the subsequent analysis. The IMU signals were high-pass filtered at 1 Hz (second-order Butterworth filter).

**Figure 1:**
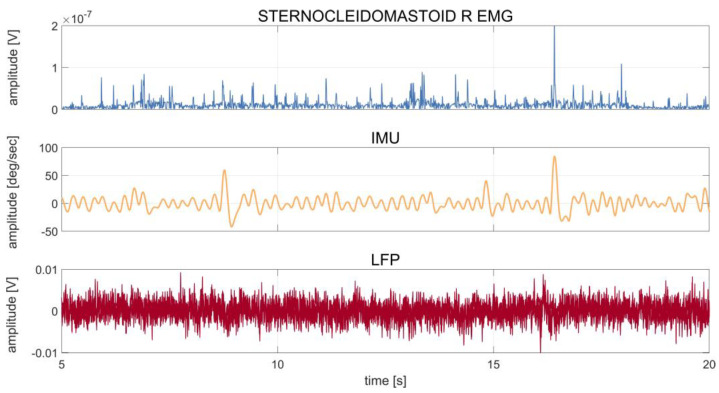
Patient DW08: time series of 20 s of recording of the right sternocleidomastoid muscle (first row), IMU (second row), and LFP (third row) during rest. The IMU time series were averaged over the three axes (x, y, z), and the LFP time series were averaged over the six contact pairs (0–3, 0–2, 1–3, left and right). The patient showed a dystonic tremor during the acquisition, as also confirmed by clinical notes and a video evaluation of the recording. The waveform of the tremor peaks captured by the IMU can also be observed in the LFPs.

Figure 1 shows an example of the synchronized recordings of EMG, IMU, and LFP in one patient (DW08).

#### 2.3.2. Spectral Analyses

The power spectral density (PSD) of all signals was computed using Welch’s method with 1-s windows and a 50% overlap.

The LFP power spectra were characterized by the presence of an aperiodic part following a 1/f power law. Following Donoghue et al. [55,59], we modeled the observed PSD as the sum of putative, periodic oscillatory components parameterized by their center frequency, power, and bandwidth, as measured from Gaussian model fits, plus an aperiodic component parameterized by the offset and slope of an exponentially decaying (1/f shaped) function. Following the parameterization procedure, we removed the 1/f aperiodic component from the PSD to analyze only the true oscillatory components. The resulting LFP power spectra were then normalized to the standard deviation of the spectrum in the range 5–95 Hz to de-emphasize spectral features prone to movement artifacts [42,60]. LFP PSDs were averaged over all contact pairs and over trials.

IMU and EMG power spectra were normalized to the standard deviation of the spectra in the range 1–64 Hz and 1–125 Hz, respectively. EMG power spectra were averaged over the four recorded muscles and over trials. IMU power spectra were averaged over the three recorded axes and over trials.

#### 2.3.3. Assessment of Tremor-Artifacts in LFP Recording and Comparison of IMU vs. EMG Regression Analysis

We investigated eventual dystonic tremor contamination in the LFP by (i) comparing PSDs across modalities and (ii) regressing multiple time-lagged copies of the EMG and the IMU out of the LFP and evaluating the effect on LFP–PSDs.

With regards to PSD comparison, individual averaged power spectra were visually inspected for peaks in the theta-alpha band (3–12 Hz), which corresponds to the range of tremor [61]. We focused on the theta-alpha frequency peaks as computed on LFP, EMG, and IMU PSDs. LFP spectra were considered contaminated by dystonic tremor when the peak in theta-alpha power aligned across LFPs, EMGs, and IMUs.

To perform the regression, all recordings were down-sampled to the lowest available sampling frequency: 128 Hz when IMUs were used, 250 Hz when EMG signals were used.

To regress the temporal dynamics of the EMG and IMU channels capturing head-tremor out of the LFPs, a temporal embedding of the EMG and IMU time series was performed. To this end, each time series *x*_*m*_(*t*) was complemented by temporally shifted versions ${\tilde{x}}_{m}$(*t*) = [*x*_*m*_(*t* + τ_1_), …, *x*_*m*_(*t* + τ_*K*_)]*^T^*, *m* = 1, …, *M*, where, *x*_*m*_(*t* + τ) was the activity of the *m*-th EMG or IMU sensor at time *t* + τ. In this work, we used K = 51 equally spaced shifts for the EMGs, ranging from τ_1_ = −250 samples to τ_K_ = 250 samples in steps of 10 samples, and K = 52 equally spaced shifts ranging from τ_1_ = −128 samples to τ_K_ = 128 samples in steps of 5 samples for the IMU. Thus, the tremor dynamics of both EMG and IMU were extracted within a window of two seconds and regressed out of LFP. The relation between the embedded signal of all M EMG and/or IMU sensors, $\tilde{x}\left(t\right)={\left[{\tilde{x}}_{1}\left(t\right),\ldots ,{\tilde{x}}_{M}\left(t\right),1\right]}^{T}$ (including an offset term) and the LFP signal $y\left(t\right)$ was modeled to be linear according to the equation $y\left(t\right)=\beta^{T}\tilde{x}\left(t\right)+y^{clean}\left(t\right)$, where $y^{clean}\left(t\right)$ denotes residual (genuine) LFP activity not explained by EMG or IMU. The (K·M+1)-dimensional vector of regression coefficients $\beta^{OLS}={\left(\tilde{X}{\tilde{X}}^{T}\right)}^{-1}\tilde{X}y^{T}$ was estimated using ordinary least-squares (OLS) regression, where $\tilde{X}=[\tilde{x}\left(1\right),\ldots ,\tilde{x}\left(T\right)]$, $y=[y\left(1\right),\ldots ,y(T)]$, and *T* denoted the number of available paired measurements of EMG/IMU and LFP activity. Using the fitted model, the part of the LFP signal that could be predicted from EMG/IMU was obtained as $\hat{y}\left(t\right)={\beta^{OLS}}^{T}\tilde{x}\left(t\right)$ [62]. The cleaned LFP signal was then obtained as the residual $y^{clean}\left(t\right)=y\left(t\right)-\hat{y}\left(t\right)$.

Cleaned LFPs were used to compute PSDs, which were compared with those obtained before performing the regression. The reduction in theta-alpha peak power after performing the regression was used as the criterion to assess the degree of contamination of LFPs by head tremor.

Because we were most interested in the theta-alpha band, we also computed the theta-alpha peak power by integrating the LFP–PSDs over a 4 Hz wide band surrounding the patient-specific theta-alpha peak (peak frequency ± 2 Hz; 4 bins) (i) without regression, (ii) with EMG regression, and (iii) with IMU regression.

#### 2.3.4. Evaluation of Pallido-Muscular Coherence with IMU-Regression

Coherence (COH) is a frequency-domain measure of the linear phase and amplitude relationships between signals. It can reveal spectrally specific functional connectivity, and it is an established method in neuroscience [63].

To investigate whether EMG and IMU carry different information about tremor, we computed COH between LFP and EMG before and after IMU regression. We postulated that the reduction in coherence after IMU regression indicates that EMG and IMU signals would carry to some extent overlapping information. As such, the LFP–EMG coherence would be due to tremor contamination, thus artifactual.

Additionally, we computed (i) LFP–IMU COH without and with IMU regression and (ii) LFP–EMG COH without and with EMG regression.

We computed coherence at frequency f as $COH\left(X,Y,f\right)=|{S_{XY}(f)|}^{2}/S_{XX}{\left(f\right)S}_{YY}(f)$, where $S_{XX},S_{YY}$ represent the auto-spectrum of signals *X* and *Y*, respectively, and $S_{XY}$ represents the cross-spectrum [57]. Note that, being already normalized, coherence spectra do not require additional normalization for across-patient analysis and do not have a prominent 1/f aperiodic component [42].

#### 2.3.5. Statistical Analysis

Statistically significant differences between (i) LFPs theta-alpha peak power with and without IMU regression, (ii) theta-alpha LFP–IMU COH with and without IMU regression, and (iii) theta-alpha pallido-muscular COH with and without IMU or EMG regression were assessed by means of a matched-pairs Wilcoxon signed rank test with a significance level of 0.05.

Significant values of pallido-muscular COH were determined by statistical comparison with a population of 1000 surrogate COH values in which any COH was destroyed. Surrogate data were obtained by randomly shifting the EMG signal 1000 times by a random offset of at least 2 s before computing the LFP–EMG COH. The significance level was set to 0.05.

## 3. Results

### 3.1. Clinical and Demographic Data

All but one patient (DW08) significantly benefited from GPi-DBS. The improvement for this patient was limited by the development of typical GPi-DBS side effects (i.e., bradykinesia). The overall average benefit from GPi-DBS was about 40% (TWSTRS score stim-off: 16.62 ± 3.85 and stim-on: 9.57 ± 4.27, mean ± standard deviation) (Table 1).

### 3.2. Tremor Contamination of LFP Recordings and Comparison of IMU vs. EMG Regression Analysis

**Figure 2:**
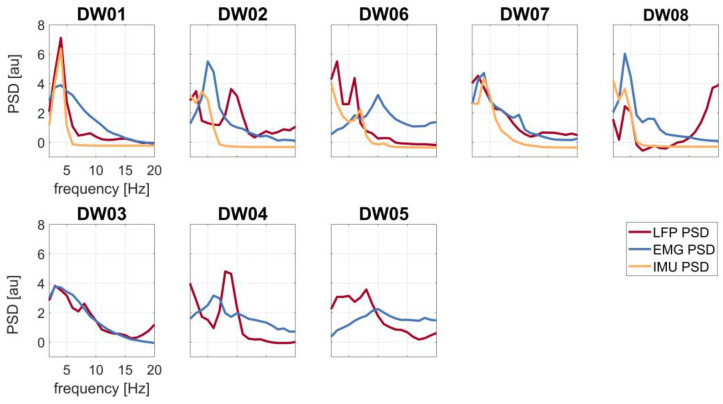
Spectral profiles of the LFP (red line), EMG (blue line), and IMU (yellow line) recordings of all patients. First row: patients with tremor (T+); second row: patients without tremor (T−).

Two patients (DW01 and DW08) showed tremor contamination of the LFP, as suggested by the alignment of the frequency peaks in the PSDs around the head tremor frequency (DW01: f = 4 Hz, DW08: f = 4 Hz) (Figure 2).

**Figure 3:**
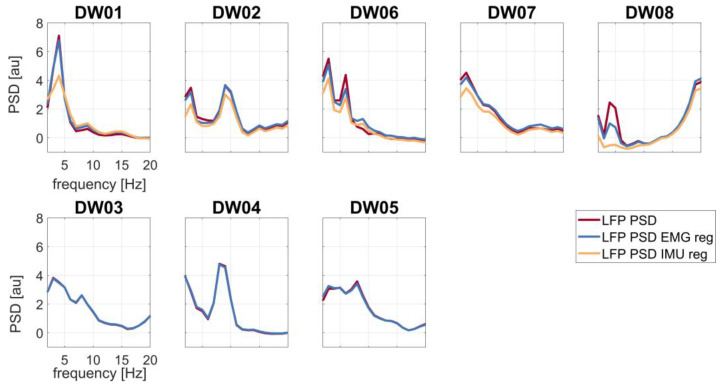
LFP spectral profiles before regression (red line), after EMG regression (blue line), and after IMU regression (yellow line). First row: patients with tremor (T+); second row: patients without tremor (T−). Abbreviation: reg (regression).

Regression-based removal of IMU signals from LFPs led to power reduction, especially in the theta-alpha band, in all T+ patients (Figure 3). Regression of the EMG signal was instead not affecting LFP theta-alpha peak power except for three patients (DW02, DW06, and DW08) (Figure 3 and Table 2).

The LFP theta-alpha peak power of each patient before and after EMG and IMU regression are shown in Table 2. IMU regression reduced the theta-alpha peak power more in comparison to EMG regression. Differences between LFPs theta-alpha peak power with and without IMU regression were statistically significant (4.66 ± 2.12 au (mean ± standard deviation)).

### 3.3. Pallido-Muscular Coherence after Cleaning

**Figure 4:**
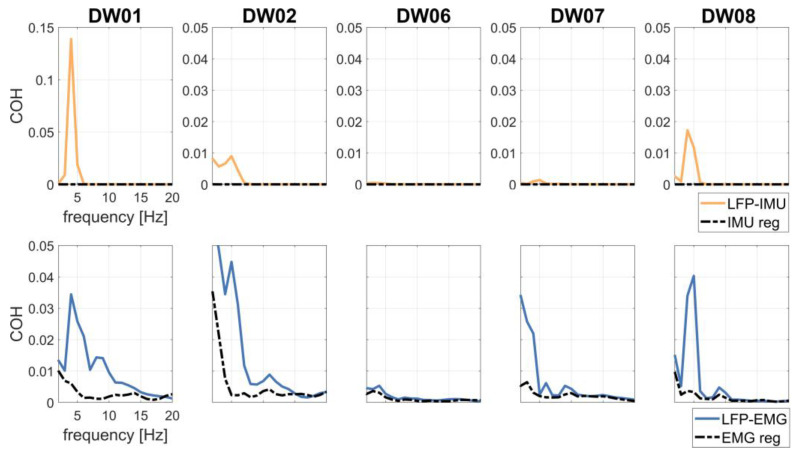
LFP–IMU COH (first row) without (continuous, yellow line) and with (dotted, black line) IMU regression in patients T+. LFP–EMG COH (second row) without (continuous, blue line) and with (dotted, black line) EMG regression in patients T+.

LFP–IMU and LFP–EMG COH profiles are shown in Figure 4. The COH drop after regression analysis was significant for both LFP–IMU COH and LFP–EMG COH.

**Figure 5:**
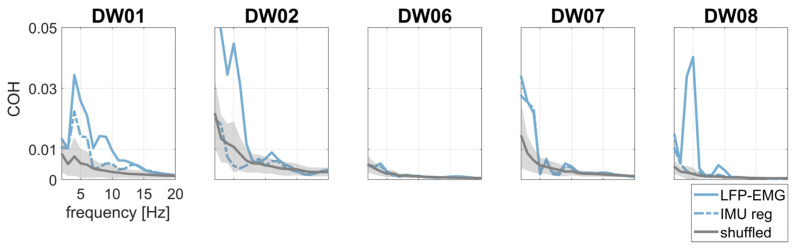
Pallido-muscular COH without (continuous, light blue line) and with (dotted, light blue line) IMU regression. Surrogate data are shown in gray as the mean (continuous line) ±2 standard deviations (shaded area).

To investigate the effects of tremor-related artifacts on pallido-muscular COH, we computed LFP–EMG COH with and without regressing the IMU out of the LFP signals. LFP–EMG COH before IMU regression was small but significant with respect to shuffled data (Figure 5). IMU regression significantly reduced pallido-muscular COH in all but two subjects (DW06 and DW07) (Table 2 and Figure 5).

### 3.4. Sensory Trick and Voluntary Rhythmic Movement

In one patient (DW02) with a clinically effective sensory trick (i.e., maneuver alleviating dystonic tremor), we computed the PSD during the sensory trick and voluntary alternating head movements with small and large amplitude. For each condition, we performed the regression of IMU and EMG.

**Figure 6:**
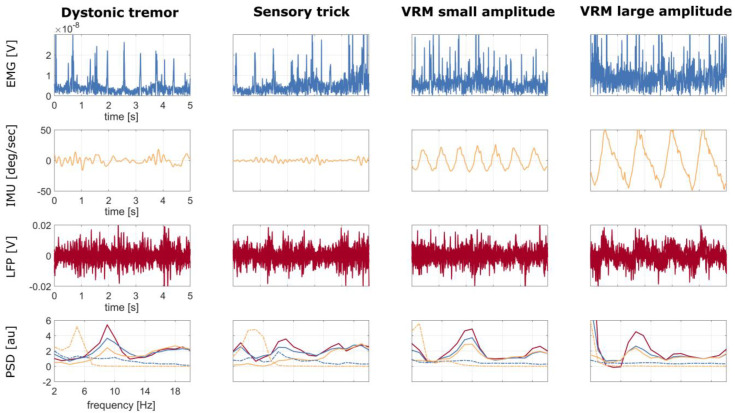
Patient DW02: time series of 5 s recordings at rest (first column), while performing the sensory trick (second column), during voluntary alternating rhythmic movement of the head with small amplitude (third column), and with large amplitude (fourth column), of the right sternocleidomastoid EMG (first row), IMU (second row), and LFP (third row) without regression. IMU and LFP time series were averaged over channels, i.e., over the three axes (x, y, z) and over the six contact pairs (0–3, 0–2, 1–3 left and right), respectively. The corresponding PSDs are shown in the fourth row: LFP without regression (continuous red line), LFP with EMG regression (continuous blue line), LFP with IMU regression (continuous yellow line), EMG (dotted blue line), and IMU (dotted yellow line). Abbreviations: VRM—voluntary rhythmic movement.

The sensory trick reduced the theta-alpha peak power in the LFP–PSDs as compared to baseline (i.e., dystonic tremor condition) (Figure 6). However, both EMG and IMU detected residual tremor activity (Figure 6), which was effectively removed from LFP–PSDs with IMU regression. Of note, the PSDs of the IMU and LFP do not show an aligned peak (Figure 6), thus suggesting that LFPs manifest harmonics of the artifactual frequency.

When looking at voluntary movements, LFP–PSDs showed movement-related artifacts at the head oscillation frequency (Figure 6).

## 4. Discussion

New DBS devices with sensing capabilities open new opportunities to improve the clinical effectiveness of DBS by optimizing stimulation parameters in response to an input signal representing symptoms, motor activity, or other behavioral characteristics [40]. Pallidal theta-alpha oscillatory activity is a promising biomarker for dystonia [45] and for future therapeutic development [46], such as improving efficacy and the timing of the therapeutic response while reducing side effects and battery consumption.

Our results show that the Percept PC is capable of recording low-frequency oscillations in chronically stimulated patients with CD but also confirm a spectral contamination due to movement artifacts (voluntary and pathological) [46].

Already, a visual inspection of the PSDs suggested artifact contamination in two subjects with dystonic tremor, with GPi-LFPs and both EMG and IMU PSDs exhibiting spectral peaks at the tremor frequency (Figure 2). When applying the IMU regression, all T+ subjects showed a reduction in power, indicating a tremor-related contamination of the raw GPi-LFP signals (Figure 3).

In our study, we showed that IMU regression was superior to EMG regression in removing the tremor-related artifact from the GPi-LFPs (Figure 3). This difference in effectiveness may be due to the greater capacity of IMUs to record a composite and irregular tremor such as dystonic tremor more distinctly [64]. Furthermore, EMG signals can be affected by skin conductance and probe placement, two aspects that increase intra- and inter-subject variability. However, we made sure that all EMG probes were adherent to the skin and placed near the bellies of selected muscles. Before electrode placement, the skin was cleaned with alcohol.

It should be noted that the EMG signal carries more information than just the kinematic (and artifactual) component of tremor, such as cross-talk from different muscles or a pathological dystonic activity [65]. To evaluate this issue, we computed pallido-muscular COH to estimate the information carried by IMU and EMG signals. After IMU regression, we observed a decrease in LFP–EMG COH at the tremor frequency in three out of five subjects (Figure 5), thus supporting the idea, in our case, of an artifactual origin of theta-alpha pallido-muscular COH.

Finally, we tested IMU and EMG-based multiple regression under different conditions, namely voluntary rhythmic head movement of small and large amplitude. This additional evaluation was performed only on the patient who showed a clinically effective sensory trick, a maneuver able to reduce tremor severity (Figure 6). We showed that both IMU and EMG regressions were effective in capturing voluntary head movements and led to a correction of GPi-LFPs proportional to the movement’s amplitude. Averna et al. [66] also showed a drastic increase in the theta frequency range during neck tilting and upper limb movement. This might refer to the higher susceptibility of some implants to contamination. Moreover, IMU regression captured the residual tremor activity present during the sensory trick maneuver that was not clinically visible. The sensory trick did not affect the rhythmic spiking of the EMG, as expected [53,67], but EMG regression led to a marginal correction of GPi-LFPs (Figure 6).

This study has some limitations. First, the limited number of patients reduced the number of recordings that were available for our analysis. Considering the low prevalence of the disease (16.3 per 100,000 [68]), the limited number of dystonic patients implanted with DBS [69], and the recent release of the Percept PC device on the market (2020), the sample size is still considerable and comparable with previous studies on dystonia [45,70]. The same limiting factors prevented the recruitment of patients with similar clinical presentations. However, we do not expect clinical features other than tremor to be relevant for the technical aims of the current work. Second, we used relatively short recordings, with lengths ranging from one to four minutes. Nevertheless, this was considered sufficient to calculate power and coherence measurements, as Popov et al. have observed good to excellent test-retest reliability of resting-state power and coherence in a large sample based on recordings that were just 100 s long [71]. Potential biases of coherence on recording length [72] were not addressed here, as all statistical comparisons were performed within subjects on data of the same length. Third, we could not record and compare tremor-free GPi-LFP recordings for the same patients. Nevertheless, we documented the performance of IMU regression in one patient with a clinically effective sensory trick. Lastly, the observed theta-alpha power reduction is not sufficient proof of the cleaning efficacy. It represents the neural activity after removal of the component that is coherent with the head tremor, which appears to introduce artifactual spectral content in this low frequency range. Although a possible desynchronization of a genuine neural component associated with tremor cannot be ruled out, this interpretation is less likely because, to the best of our knowledge, dystonic tremor has not been associated with any oscillatory activity of the LFPs to date [47].

## 5. Conclusions

LFP-based biomarker detection will become standard in clinical practice, thus enabling better understanding and monitoring of distinctive neural signatures associated with specific symptoms or behaviors. Pallidal theta-alpha oscillations may be critical for understanding the pathophysiology of dystonia. They could act as a useful biomarker not only for programming stimulation parameters but also for adaptive DBS. However, critical use of newly available technologies is necessary to address possible drawbacks. Our work suggests caution when considering LFP recordings, as they may be susceptible to contamination by movement artifacts. This is particularly the case for head tremor in dystonia but also applies to patients with repetitive involuntary movements such as essential tremor, Tourette syndrome, or Parkinson’s disease. Neglecting this contamination can lead to misinterpretation or hiding significant findings. We here provide methodological guidance on how to clean LFP recordings from head tremor artifacts. Regressing out head motions concurrently recorded with IMU might substantially alleviate LFP contamination and facilitate the neurophysiological interpretation of LFP analyses.

## Figures and Tables

**Table 1:** Demographic and clinical data. Abbreviations: T+—patients with head tremor; T−—patients without head tremor; CD—cervical dystonia; MFD—multifocal dystonia; DM—dystonia with myoclonus; TWSTRS—Toronto Western Spasmodic Torticollis Rating Scale; DBS—Deep Brain Stimulation; NA—not available. * Identifies patients studied at battery replacement. ** Age at battery replacement.

Patients		DW01 (T+)	DW02 (T+)	DW06 (T+)	DW07 * (T+)	DW08 (T+)	DW03 * (T−)	DW04 * (T−)	DW05 (T−)
Sex		F	F	F	F	F	M	M	F
Age		52	44	57	50	33	74	62	65
Age at onset, years		38	36	42	Childhood	Childhood	42	Childhood	Childhood
Age at surgery		50	43	56	48 **	31	72 **	62 **	63
Disease		CD	CD	CD	MFD	DM	CD	DM	DM
TWSTRS pre-DBS, score		18	19	13	NA	22	22	NA	18
TWSTRS post-DBS, score	Stim-off	16	18	8	17	19	16	21	18
TWSTRS post-DBS, score	Stim-on	15	14	6	8	NA	5	13	6

**Table 2:** Columns 2–4: theta-alpha peak power [au] without and with EMG or IMU regression. Differences (Δ) between LFP theta-alpha peak power without and with EMG or IMU regression are shown in brackets. Columns 5–6: averaged theta-alpha pallido-muscular COH without and with IMU regression. The differences (Δ) between LFP and EMG COH without and with IMU regression are shown in brackets. Abbreviations: COH—coherence; EMG—electromyographic recording; IMU—inertial measurement unit recording; LFP—local field potential; reg—regression; NA—not available.

Patient	LFP θ-α Power	LFP θ-α Power EMG Reg (Δ)	LFP θ-α Power IMU Reg (Δ)	LFP-EMG θ-α COH	LFP-EMG θ-α COH—IMU Reg (Δ)
DW01	17.84	17.93 (−0.09)	15.01 (2.83)	0.015	0.008 (0.007)
DW02	8.16	7.03 (1.13)	4.50 (3.66)	0.020	0.007 (0.013)
DW06	14.34	12.85 (1.49)	9.78 (4.56)	0.002	0.002 (0)
DW07	14.56	13.70 (0.86)	10.59 (3.97)	0.007	0.007 (0)
DW08	6.07	2.66 (3.41)	−2.21 (8.28)	0.010	0.002 (0.008)
DW03	16.32	16.22 (0.10)	NA	NA	NA
DW04	11.06	10.88 (0.18)	NA	NA	NA
DW05	7.33	8.00 (0.67)	NA	NA	NA

## Data Availability

The data presented in this study are available upon request. The data are not publicly available due to privacy reasons. Inquiries can be filed to the corresponding author (Jasmin Del Vecchio Del Vecchio, University Hospital Würzburg, Department of Neurology; Josef-Schneider-Straße 11, 97080 Würzburg; phone: +49-(0)931/201-23605; fax: +49-(0)931/201-24901; E-Mail: delvecchio_j@ukw.de).
